# Spontaneous expulsion of a huge appendiceal fecalith after endoscopic treatment

**DOI:** 10.1055/a-2578-2400

**Published:** 2025-04-29

**Authors:** Fan Wang, Hongling Wang, Sikai Chen, Qiu Zhao, Jiali Hu

**Affiliations:** 1 Department of Gastroenterology, Zhongnan Hospital of Wuhan University, Wuhan, China; 2 Department of Ultrasound, Zhongnan Hospital of Wuhan University, Wuhan, China; 3 Department of Pathology, Affiliated Hospital of Jiujiang University, Jiujiang, China

A 38-year-old woman was admitted due to intermittent right lower abdominal pain experienced for half a year. At the local hospital, abdominal computed tomography (CT) scan showed a huge appendiceal fecalith (1.35 × 0.81 cm) ( Fig. 1 ). After admission, abdominal ultrasonography confirmed the appendiceal fecalith (1.49 × 0.58 cm; 3.3 cm from the orifice; appendix size 6.7 × 0.7 cm, retroileal location; diameter 0.8 cm; wall thickness 0.12 cm). Endoscopic retrograde appendicitis therapy using an appendoscope (eyeMAX, 9-French; Micro-Tech [Nanjing] Co., Ltd., China) was planned 1 .

**Fig. 1:**
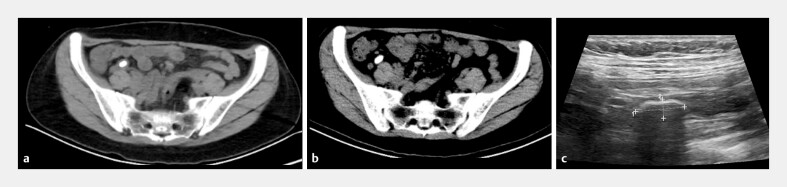
A huge appendiceal fecalith was detected on imaging. **a** Computed tomography (CT) scan half a year before endoscopic treatment. **b** Repeat CT scan before endoscopic treatment. **c** Ultrasonography before endoscopic treatment.

During the operation, the appendoscope was inserted into the appendiceal lumen, and the mucosa was smooth ( Fig. 2 , Video 1 ). Lumen stenosis was detected, and a guidewire was used for exploration before dilating the stenosis repeatedly with the appendoscope body. The fecalith was found at the end of the appendix but could not be grasped with a basket (diameter 1.0 cm). Finally, a plastic stent (8.5 Fr × 5 cm) was implanted into the appendix from the ileocecum.

**Fig. 2:**
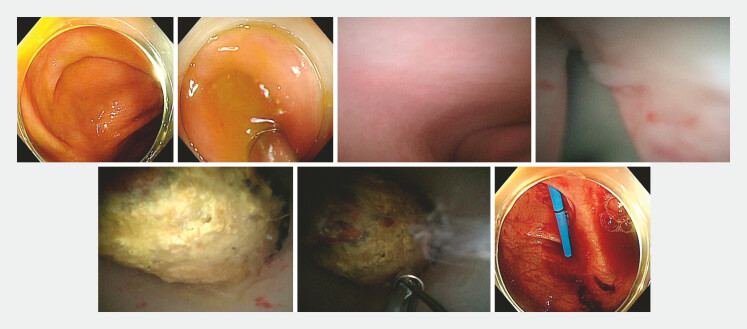
Endoscopic treatment of the appendiceal fecalith. The appendoscope was passed through the appendiceal orifice and stenosis with the help of a guidewire. The fecalith was detected but could not be grasped with a basket. A plastic stent was placed.

Spontaneous expulsion of a huge appendiceal fecalith after endoscopic treatment.Video 1

Right lower abdominal pain was noted during the following 3 days. On the 4th day, the patient’s pain was significantly relieved, and simultaneous CT scan showed expulsion of the appendiceal fecalith into the sigmoid colon ( Fig. 3 ). On the 11th day, the stent was expelled with the stool. To the best of our knowledge, this is the first reported spontaneous expulsion of a huge appendiceal fecalith after endoscopic treatment.

**Fig. 3:**
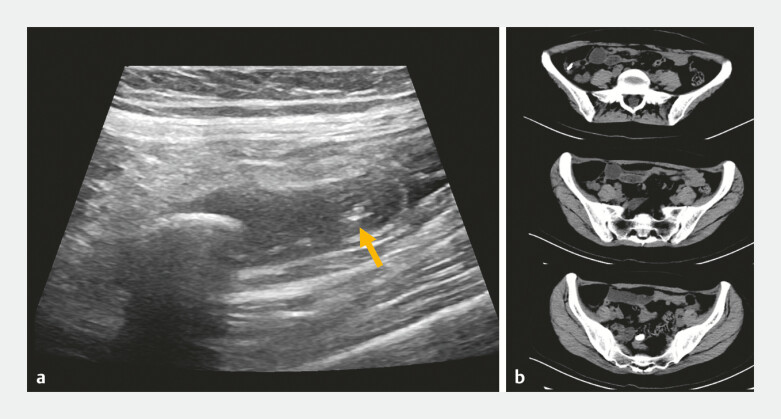
Imaging after endoscopic treatment. **a** Ultrasonography detected the fecalith (1.57 × 0.64 cm) and stent (arrow) 2 days after endoscopic treatment. **b** Computed tomography scan detected the stent end around the appendiceal orifice and fecalith expulsion into the sigmoid colon (size 1.46 × 0.86 cm) 4 days after endoscopic treatment.

Endoscopy_UCTN_Code_TTT_1AQ_2AF

## Notes

Endoscopy E-Videos https://eref.thieme.de/e-videos
*E-Videos* is an open access online section of the journal *Endoscopy* , reporting on interesting cases and new techniques in gastroenterological endoscopy. All papers include a high-quality video and are published with a Creative Commons CC-BY license. Endoscopy E-Videos qualify for HINARI discounts and waivers and eligibility is automatically checked during the submission process. We grant 100% waivers to articles whose corresponding authors are based in Group A countries and 50% waivers to those who are based in Group B countries as classified by Research4Life (see: https://www.research4life.org/access/eligibility/ ). This section has its own submission website at https://mc.manuscriptcentral.com/e-videos .
